# Physical environment research of the family ward for a healthy residential environment

**DOI:** 10.3389/fpubh.2022.1015718

**Published:** 2022-10-13

**Authors:** Yuqing Zhang, Xiao Liu, Qinglin Meng, Bin Li, Luca Caneparo

**Affiliations:** ^1^School of Architecture, South China University of Technology, Guangzhou, China; ^2^Department of Architecture and Design, Politecnico di Torino, Torino, Italy; ^3^State Key Laboratory of Subtropical Building Science, South China University of Technology, Guangzhou, China; ^4^Architectural Design and Research Institute Co., Ltd., South China University of Technology, Guangzhou, China; ^5^Faculty of Architecture, The University of Hong Kong, Hong Kong, Hong Kong SAR, China

**Keywords:** physical environment, healthy building, indoor environment quality, family ward, aging, healthy residential environment

## Abstract

Climate change and population aging are two of the most important global health challenges in this century. A 2020 study by the Environmental Protection Agency showed that average people, particularly older adults, spent 90% of their time at home. This is even more evident during the coronavirus disease 2019 (COVID-19) pandemic. Home-based care models have become a new trend. The health and comfort of the living environment profoundly impacts the wellbeing of older adults. Therefore, research on the physical environment of the family wards has become an inevitable part of promoting the health of older adults; however, current research is still lacking. Based on the study and analysis of continuous monitoring data related to elements of the physical environment (thermal comfort, acoustic quality, lighting quality, and indoor air quality) of family wards, this paper explores the living behaviors of the participants in this environmental research (open or closed windows, air conditioning, artificial lighting, and television) on the indoor physical environment. (1) While referring to the requirements of international standards for an indoor aging-friendly physical environment, we also discuss and analyze the physical environment parameter values according to Chinese standards. (2) People's life behaviors have different degrees of influence on the elements of indoor physical environments. For example, opening doors and windows can alleviate the adverse effects of indoor environmental quality on the human body better than simply turning on the air conditioner. (3) Owing to the decline in physical function, older adults need special care. Studying the status quo of physical environmental elements and proposing suitable environmental improvement measures for aging are of great significance. (4) This research aims to address global warming and severe aging and to contribute to sustainable environmental development.

## Introduction

In the face of climate change and increasing aging, China's home quarantine policy in response to the coronavirus disease 2019 (COVID-19) has forced older adults to contend with even more significant challenges in seeking medical care. China entered the state of an aging society at the end of the 20th century, and the degree of aging has continued to deepen. Climate change and population aging are two of the most critical global health challenges of the 21st century. According to the Population Division of the United Nations Department of Economic and Social Affairs (1), in 2050, China's over-60 population will account for 23.8% of the total population. China is promoting the concept of “home-based care” or “in-place care” through adaptation and transformation to encourage older adults to obtain higher-quality living conditions in a healthy and sustainable living environment (2–4). A 2020 study by the Environmental Protection Agency shows that average people, particularly older adults, spent 90% of their time at home. This is even more evident during the COVID-19 pandemic. Another study also showed that older adults spend ~22 h a day at home, mainly in bedrooms and living rooms (5–9). However, little is known about the physical environment, closely related to wellbeing and living healthily in older adults (10–12). The World Health Organization (WHO) proposed an active aging framework, with safe housing for older adults as a critical theme in the physical environment dimension (13). Creating age-friendly environments is a strategic objective in the World Health Organization's (14). Global Strategy and Action Plan on Aging and Health (2016–2030). It also relates to many of the United Nations' (UN) (15). Sustainable Development Goals and the European Union' (EU) new Smart Healthy Age-Friendly Environments (2019–2023) policy development program as well as the recently launched Center for Active Aging and Innovation established by the Association of Southeast Asian Nations (16).

With improvements in living standards, human health and environmental quality have received widespread attention from researchers. China's carbon neutrality challenge reveals the increasingly apparent negative impact of human construction activities on the environment (17, 18). Sustainable development is a global consensus that promotes the healthy development of society, with more environmental issues involved increasingly (19). The built environment accounts for 40% of the world's annual final energy (20), with residential buildings accounting for a significant proportion. Environmental sustainability is a growing concern in developing the living environment, driven partly by calls for sustainable and eco-friendly lifestyles (21–24). Therefore, the need to create green living environments for residents, especially older adults, by incorporating a healthy environment is increasing (25). Older adults are essential contributors to environmental sustainability (26). Occupants often cope with environmental discomfort by adapting to it or adapting themselves (27). The role of adaptive behavior in improving occupant comfort and environmental quality as well as in improving occupant satisfaction has been confirmed by scholars (28–31). Occupant behavior is a significant source of uncertainty in building performance (32–34). Different disciplines and fields of work, from health to urban planning, social care, and information technology (35–37), recognize the value of involving occupants in environmental design.

Family wards that meet and safeguard the health needs of older adults must ensure a sustainable, safe, and comfortable indoor physical environment (38). Some scholars have explored the relationship between the living environment of older adults and physiological and psychological factors (39). Others have concluded that the thermal and acoustic environment has a more critical impact on the overall indoor physical environment quality than that on indoor air quality (40, 41). Moreover, research has shown that the four elements of the physical environment have different degree of importance in the existing standards for assessing and certifying residential indoor building environments (42). Other scholars have compared older adults' thermal comfort with the current predicted mean vote (PMV) comfort models and concluded that their thermal sensitivity is lower than the sensitivity level of the PMV model used in many standards (43). They found that older adults were more tolerant than non-older adults and preferred higher temperatures (44). The thermal behavior and living conditions of older adults (45) in naturally ventilated dwellings (46, 47) have also been reported.

After considering the wellbeing and physical and mental health of older adults, there is a consensus on developing a high-quality residential environment for them (48). A two-way link exists between the built environment and human behavior, health, and wellbeing (49). The quality of the indoor physical environment depends on the indoor environmental performance of the building as well as human behavior (50). Environmental gerontological theories suggest that indoor physical environment is a crucial factor affecting the wellbeing of older adults (51). Therefore, understanding the complex interactions between humans and the indoor built environment (52) and how the behavioral activities of occupants affect the quality of the indoor physical environment is crucial (53, 54). The current study considered different combinations of physical environmental factors, such as the thermal and acoustic environments, light and acoustic environments, light environment and indoor air quality, and acoustic environment and indoor air quality. Researchers are most interested in the effects of dependent variables, such as occupant comfort, feelings, preferences, and satisfaction (55–57). For example, scholars have assessed the extent to which window opening/closing behavior is driven by outdoor climatic conditions, indoor air quality, or other parameters. Studies have concluded that indoor and outdoor air temperature, indoor air quality, and solar radiation are the main drivers of occupant control of window opening and closing (58, 59). Some scholars have proved *via* experimental approaches that the clothing of occupants and the interaction between people and the built environment affect behavior patterns, impacting the physical environment (60). Occupant interaction with the thermostat affects energy consumption and the quality of the indoor physical environment. The thermal adaptation behavior of occupants includes adjusting fans, heaters, and thermostats when they feel uncomfortable (61). Humans generally adapt to indoor physical environments through behavioral, physiological, and psychological adaptations (62); for example, by opening or closing windows and curtains, the thermal and light environments in the physical environment elements can be regulated (63), including increasing indoor airflow (64). Another study found that occupants used ceiling fans much more than air conditioning systems and chillers to regulate a home's physical environment (65). Other studies have shown that window regulation is related to environmental factors such as temperature and seasonal changes, size, and distance to windows. However, it should be noted that while such measures improve occupants' comfort, they can also lead to a waste of energy (66). Therefore, scholars should find a balance among the various comfort-related factors (67).

Based on research on the indoor physical environment of a family ward in Guangzhou, China, this study focuses on the correspondence between occupant behavior and elements of the physical environment, analyzes the parameters of the physical environment suitable for aging, and proposes measures to optimize the indoor physical environment of family wards. This research aims to solve the problems of aging and the sustainable development of the living environment. Following existing standards, we measured the indoor physical environment quality to evaluate the relationship between residents' preferences and the physical environment in family wards. Our conclusions should be applied to existing buildings (to assess their present status), retrofit projects (to evaluate them before and after renovation), and new facilities (for design and benchmarking).

## Research objects

### Family ward

**Figure 1:**
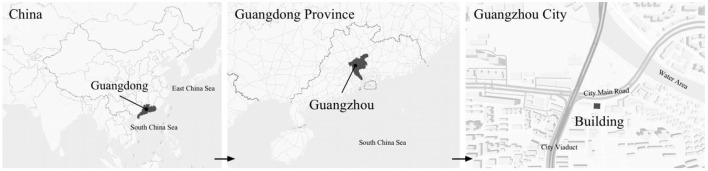
Location characteristics of experimental building.

The family ward is located in the city of Guangzhou, China. Guangzhou has a southern-subtropical maritime monsoon climate. Because it is located close to the South China Sea and is affected by warm and humid tropical marine air masses, it has distinct climate characteristics. Guangzhou generally has hot summers (June–August) and relatively mild winters (December–February). The building where the family ward is located is close to where two main roads of the city intersect (Figure 1). A healthy female experimenter with a height of 1.6 m was selected for this study. The experimenter observed the changing aspects of the indoor physical environment by simulating activities of daily living in the family ward environment controlled by older adults or individuals with disabilities. This study measured the light, acoustic, and thermal environments as well as the indoor air quality of the four main activity areas of the family ward. These activity areas primarily consisted of the reception area, rest area, passing hall, and bathroom. The total area of the family ward was 26 m^2^.

**Figure 2:**
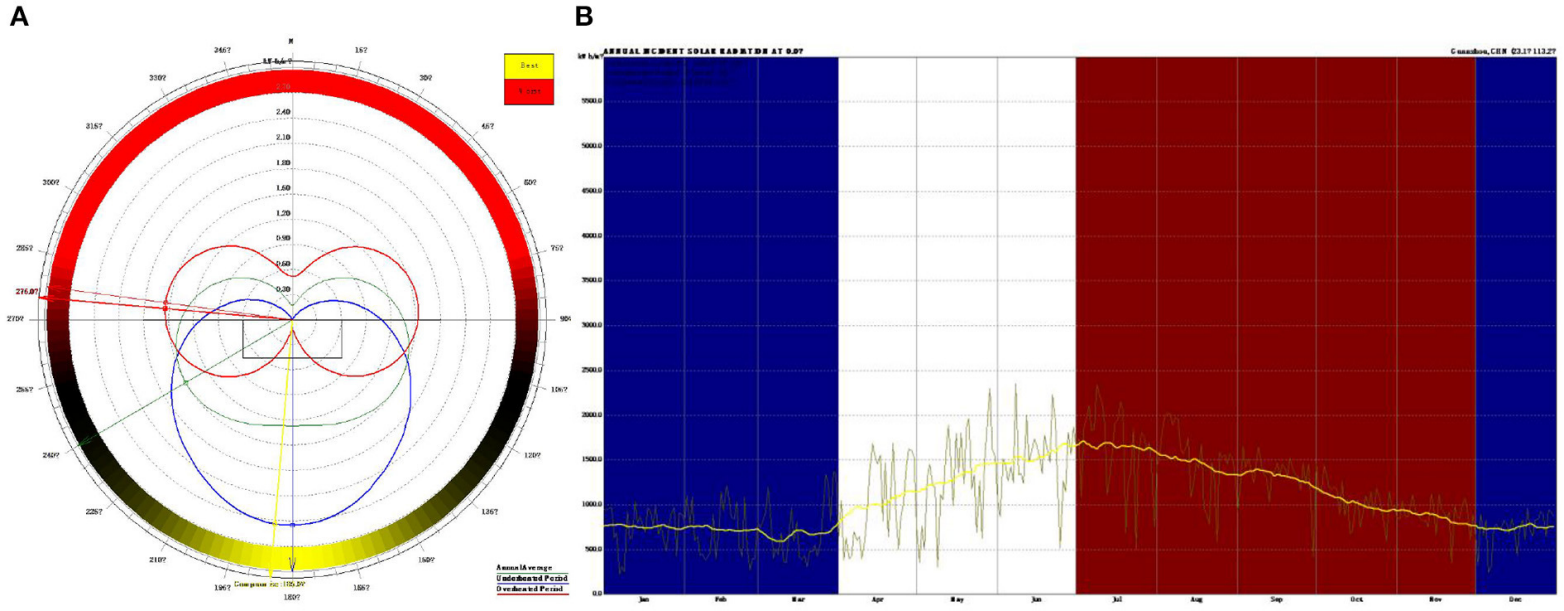
Analysis results obtained by the weather tool. **(A)** Solar radiation; **(B)** The overheating period and the supercooling period.

We chose the Weather Tool (Autodesk Analysis Ecotect software) to analyze the physical environment of the family ward area. According to our analysis of Guangzhou meteorological parameters, we were able to determine the family ward's overall environmental conditions. The analysis results of the meteorological tools can assist in verifying the reliability of the measured results and provide preliminary data for analyzing the impact of the outdoor environment on the indoor environment. According to the analysis results obtained using the Weather Tool, the annual average amount of solar radiation in the Guangdong area is primarily received in the direction of West South by 60°, the subcooling period is concentrated in the South direction, and the overheating period is focused on the North West by 5° (Figure 2A). The Figure 2B shows that the supercooling period in the blue area is from late November to early April. In this study, the experimental subjects in the family ward sat north and faced south, and the measured time was during the supercooling period. The Guangdong area, where the family ward is located, has low solar radiation intensity and low heat from direct sunlight. The outdoor cold air radiation adversely affects the indoor thermal environment. Based on the above analysis results and other constraints, we selected December 13–15, 2021, as the test time for the supercooling period.

### Physical environment

The indoor micro-environment performance of family wards can be assessed in terms of thermal comfort, lighting quality, acoustic quality, and indoor air quality, generally and collectively referred to as the indoor physical environmental quality (IPEQ). The IPEQ of family wards is also affected by outdoor sources, building characteristics, and indoor pollutants. Physical environment comfort is usually defined as “that condition of mind that expresses satisfaction with the physical environment.” Providing comfort indoors is a fundamental objective of architects, engineers, and the allied building sector professions.

#### Thermal comfort

Recently, an increasing number of researchers have devoted themselves to investigating and analyzing the indoor thermal environment of residential buildings (68, 69). The impact of thermal environments on the emotional wellbeing of occupants is complex (70). The indoor thermal comfort of an indoor environment can be estimated using a thermal adaptation model based on the currently applicable standards the American society of heating, refrigerating and air-conditioning engineers, Ins. (ASHRAE) 55–2020 and the energy performance of buildings (EN) 16798–2019. Thermal comfort is a psychological and physiological condition that expresses a human's perception of the temperature, humidity, and wind environment of their surrounding indoor and outdoor environments (71). Objective factors of environmental quality include air temperature, the average radiant temperature of the surrounding surfaces, relative humidity, and wind speed (72). Thermal comfort significantly impacts occupant health, particularly the perception of household indoor environmental quality, which is especially important for vulnerable groups such as older adults (73).

Since the invention of air conditioners in the early 20th century, people have become accustomed to manually controlling indoor climates. Despite continuous improvements in control technology, room temperature has remained the dominant control variable in air-conditioning technology for over a century. Research has shown a definite link between occupants' exposure to low or high indoor temperatures and their health. Relevant organizations and documents outlined indoor temperature range limits. In 1987, the world health organization (WHO) guidelines in indoor temperatures recommended indoor temperatures be maintained at 18°C, or 20–21°C in rooms used by older adults (74). Several other relevant studies suggest that indoor temperatures should be close to 25°C in the absence of physical activity (75) and should be lowered but maintained above 20°C. In addition, according to T18883-2002, the standard temperature for heating in winter should be in the range of 16–24°C, and the typical temperature for air conditioning in summer should be 22–28°C (76).

Exposure of sensitive individuals to low temperatures can lead to decreased resistance to respiratory infections and increased blood pressure. Thermal fatigue and heat stroke can occur when sensitive individuals are exposed to high temperatures. The risks are thus substantial for older adults. Scholars have presented different research results regarding the thermal comfort of older adults. Relevant research shows that physically and psychologically, older people prefer a warm environment. Thermal sensation of older adults is general 0.5 scale units (on a 7-point thermal sensation scale, Table 1) lower than thermal sensation of younger adults (77). Other scholars have also proved that during the heating period, controlling the indoor temperature within the range of 22–26°C has a positive impact on the health and comfort of occupants, particularly older adults, and helps improve the quality of the indoor environment (78).

**Table 1:** The thermal comfort index PMV and its relationship to thermal sensitivity (96).

**−3**	**−2**	**−1**	**0**	**+1**	**+2**	**+3**
Cold	Cool	Slightly cool	Neutral	Slightly warm	Warm	Hot

Thermal sensation votes were recorded using the American Society of Heating, Refrigerating, and Air-Conditioning Engineers (ASHRAE) 7-point scale of thermal sensation (97), with questions ranging from hot (+3) to cold (−3) (98).

The temperature and humidity are critical indicators of the comfort of a room. Indoor humidity is vital to human thermal comfort, indoor air quality, and feelings of dryness. Some scholars have argued that the humidity level should be maintained between 25 and 55%. The humidity limit is 12 g/kg in ASHRAE 55-2013, 40–70% in the chartered institution of building services engineers (CIBSE) guide A: environmental design (79), and I: 30–50%, II: 25–60%, III: 20–70%, and <12 g/kg in EN (European Committee for Standardization) 15251 (80). Owing to differences in climate, the living habits of residents, and older adults' physical fitness in different countries and regions, the results obtained by scholars vary. The thermal environment parameters obtained by researchers were controlled within a clear limit range. Considering that older adults constitute a vulnerable group, the discussion of thermal environment parameters requires further case support.

**Figure 3:**
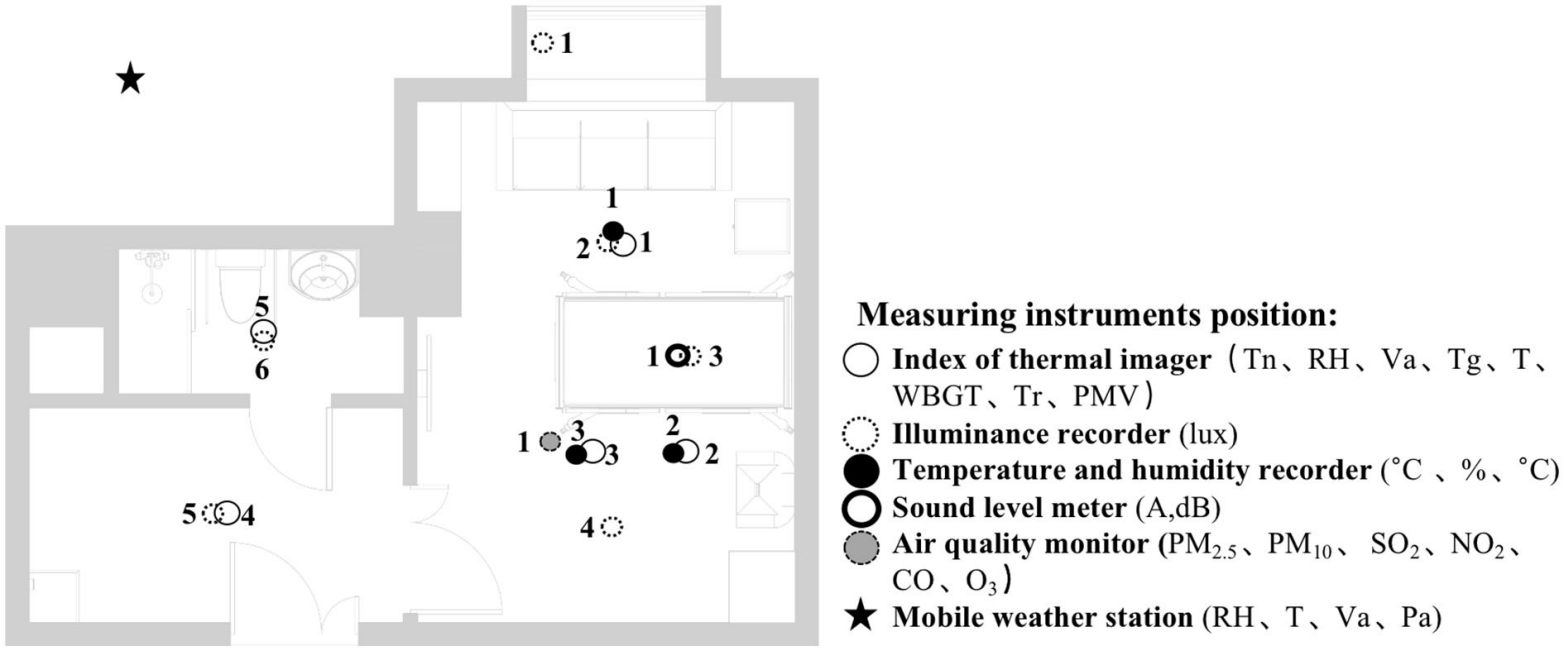
Instruments' position plan.

The indoor thermal environment includes the relative humidity (RH), indoor airflow velocity (Va), earth thermometer temperature (Tg), predicted mean votes (PMV), and many other elements. In winter, the Va is usually small, and the average radiant and air temperatures have a greater impact on human thermal comfort. In addition to taking temperature and humidity measurements, the measurement of the indoor physical environment also includes the average radiant temperature of the room. In this study, the mean radiant temperature was approximated as the area-weighted average of each surface temperature. To present the research results more intuitively, a thermal imager was selected for the visual recording in this study. The subjects here had a metabolic rate of 1.2 met and a thermal resistance of 1 clo of clothing. The area where occupants often stay and three measurement points near the hospital bed were selected. The location of selected points are shown as the temperature and humidity measurement point location in Figure 3.

#### Lighting quality

One of the most direct environmental factors affecting the comfort of older adults is the lighting intensity in the room. Occupants' requirements regarding their lighting environment change with age owing to physiological changes. Research shows that vision changes are among the most important physical changes that occur with aging (81, 82). Compared to non-older adults, older adults require higher illumination levels, especially short-wavelength light, to experience visual and circadian effects (83).

A Japanese company's study showed that the overall comfort level of a room for older adults varies between 50 and 250 lx. The comfortable illuminance value of young people accounts for ~2/3 of this illuminance. The findings of this study suggest that older adults have a greater need for lighting (81, 84, 85). China's “Architectural Lighting Design Standard GB50034-2013” stipulates the standard value of lighting at different heights of residential buildings. For example, at a level of 0.75 m, in an older adult's bedroom, the general activity area should meet a standard illuminance value of 100 lx, and the bedside and reading areas should complete the mixed lighting of 300 lx. In an older adult's living room, general activity should meet a 200 lx standard and a mixed illuminance standard of 500 lx for writing and reading. For the bathroom, an illuminance of 100 lx is required. According to China's “architectural lighting design standard GB50033-2013,” the natural light intensity of bedrooms and living rooms in residential buildings should be at least 300 lx.

The following conclusions can be drawn from the above analysis combined with the existing standard requirements. A bedroom area with a partial reading function and mixed lighting should meet the requirements of 100–300 lx, and the natural lighting should meet the requirements of at least 300 lx. A living room needs to meet the mixed lighting standard value of 200 lx and natural lighting illuminance value of 300 lx. An auxiliary hall must meet the requirements of 100 lx mixed lighting intensity. Finally a bathroom must meet the illuminance requirement of 100 lx for mixed lighting.

#### Acoustic quality

According to the “Code for Sound Insulation Design for Civil Buildings GB20118-2010,” the regulations on the allowable noise level (A sound level, dB) for residential buildings with higher requirements are as follows: bedroom daytime ≤45 dB (A), nighttime ≤37 dB (A); living room all day ≤45 dB (A). According to the “Acoustic Environment Quality Standard GB3096-2008,” acoustic environment functions are divided into five types. Type 0 refers to areas requiring special quietness such as rehabilitation and recovery areas. The family ward in this study can be considered this type of area and requires a higher standard of improvement and control of indoor environment quality. For the Type 0 sound environment functional area, the environmental noise limit was ≤50 dB (A) during the day and ≤40 dB (A) at night. According to the Chinese Industry Standard “Architectural Design Standards for Elderly Facilities JGJ450-2018,” when the occupants are older adults, the interior should have good sound insulation and noise reduction devices. The noise of the indoor living environment (86) should be <40 dB (A), and the air sound insulation should not be >50 dB (A). The impact sound should not exceed 75 dB (A) (87). Older adults are more tolerant of sound, but they are also more sensitive. Excessive and unnecessary noise can harm older adults' health and hinder their recovery from hearing loss. Long-term exposure of older adults to noise above 65 dB (A) can cause serious health problems, such as sleep disturbances, hearing loss, tinnitus, hypertension, and cardiovascular disease. According to relevant Chinese standards, for rooms with a residential building area of <30 m^2^, the measuring point is the center of the room. The family ward used in this study was 26 m^2^. Therefore, the center point of the room was selected to measure the acoustic environment parameters. The area is also an activity area for the occupants. The measurement point was 1.2 m away from the ground and 1.0 m away from the indoor wall.

#### Indoor air quality

The indoor air quality standards have improved over the past few years. Indoor air quality can have a wide range of effects on occupants' health and immune systems. The cleaner the indoor air, the more resistant the occupants are to viruses and infections. In winter, occupants are more likely to move indoors, shortening the distance among them, and increasing their risk of disease. According to the European environment agency (EEA), air pollution is Europe's most significant environmental health risk, especially in urban areas. Particulate matter (PM), nitrogen dioxide (NO_2_), and ground-level ozone (O_3_) cause the most significant damage, leading to ~400,000 premature deaths annually.

This study primarily refers to the regulations on indoor environmental parameters during the winter heating period in China's “Indoor Air Quality Standard GB/T18883-2002.” Regarding physical parameters, the indoor temperature was set to 16–24°C; the relative humidity was controlled at 30% to 60%, and the air volume was 0.2 m/s. The indoor chemical parameters were as follows: the sulfur dioxide (SO_2_) concentration should be <0.5 mg/m^3^, the nitrogen dioxide (NO_2_) concentration should be controlled at 0.24 mg/m^3^, the carbon monoxide (CO) concentration should be <10 mg/m^3^, and the inhalable particles 10 (PM_10_) concentration should be controlled at 0.15 mg/m^3^. According to standard requirements, when the room area is <50 m^2^, 1–3 sampling points are selected as the setting. The sampling point should not be near the ventilation opening, and the distance from the wall should be more than 0.5 m. The instrument should be set at a height of 0.5 m to 1.5 m. According to the standard requirements, the doors and windows were closed for 24 h before the measurement in this experiment.

## Methods

### Parameters and instruments

A portable Delta Ohm HD32.2 instrument and a thermal imager were used to measure the thermal environment. The instrument mainly measures indoor thermal environment parameters and records data every minute. The parameters include Air temperature (Ta), RH, mean radiation temperature (Tr), natural wet-bulb temperature (Tw), Tg, and wind speed (WS). This study used Tg, Tw, and Ta to estimate a composite temperature index and the wet bulb globe temperature (WBGT), following the International Organization for Standardization (ISO) standard. These measurements were then used to evaluate the influence of temperature, humidity, and solar radiation on people. The most commonly used heat balance estimates PMV model was utilized to evaluate the thermal comfort of the family ward in this study. The PMV model was initially developed by Fanger based on indoor experiments to establish a thermal balance model for the thermal comfort of air-conditioned buildings (88). Fanger and Toftum (89) was among the first to study the parameters affecting indoor environmental quality. Other researchers have since validated its applicability to naturally ventilated buildings (42, 90, 91). In this study, the thermosensory scale from the PMV model (from 3 cold to + 3 hot) was obtained from the ASHRAE Standard 55 (1992, 2013, 2020; Table 1) (92–94). They were also combined with Ta, Tr, WS, HR, occupants' activity level (met), and occupants' clothing insulation (Clo) to determine the thermal comfort of the family ward.

In this study, the room acoustic environment parameter, the A-weighted sound pressure level, was recorded every minute. A portable sound level meter AWA5633 instrument and the NoiseLab-Lite mobile application were calibrated for each other. The sound pressure level measured by the A-weighting network was expressed as LA in dB (A).

This study used a portable ONSET MX1104 instrument to measure indoor lighting environment parameters (illuminance value, lux). The temperature and humidity at the corresponding measuring points were also recorded. The data form a contrasting condition designed to reduce measurement errors due to sensor drift (data are recorded every minute).

To obtain relevant data on indoor environmental quality parameters, this study used a portable Sniffer4D Mapper instrument to record indoor air quality parameter data, such as SO_2_ (μg/m^3^), CO (μg/m^3^), NO_2_ (μg/m^3^), PM_2.5_ (Particle size is 0.3~2.5 μm, μg/m^3^), PM_10_ (Particle size is 0.3~10 μm, μg/m^3^), every minute. Air pollution is a recognized risk factor for cardiovascular and respiratory diseases. In this study, existing instruments were used to detect and analyze indoor environmental quality parameters as much as possible.

The indoor and outdoor environments are closely related, and the outdoor climate causes periodic changes in the indoor environment (95). In this study, we selected the 2000 series WatchDog mobile weather station to monitor the outdoor physical environment quality of the family ward.

The monitored meteorological parameters mainly included temperature, relative humidity, wind speed, wind direction, and rainfall. The weather station was installed on the open roof of the building and coincided with the time measured in this study, December 13–15, 2021. All instruments and their corresponding physical environment parameters for the family ward are listed in Table 2.

**Table 2:** Basic characteristics of the experimental instruments.

**Environmental parameters**	**Instruments**	**Measuring range**	**Accuracy**	**Time interval/min**	**Experimental instruments**
Thermal comfort	Delta Ohm HD32.2	−40 to 100°C (TP3207.2, dry-bulb temperature)	Classe 1/3 DIN	1 min	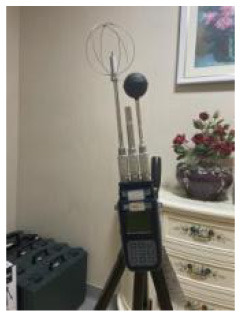
		−10 to 100°C (TP3276.2, black-bulb temperature)			
		4 to 80°C (HP3201.2, natural wet-bulb temperature)	Class A		
Acoustic quality	AWA5633 and NoiseLab-Lite mobile application	35 dB−130 dB (A)	2 level	1 min	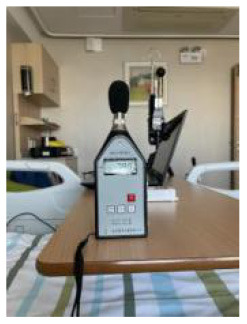
Lighting quality	ONSET MX1104	0–167,731 lux (lighting)	±10%, direct sunlight	1 min	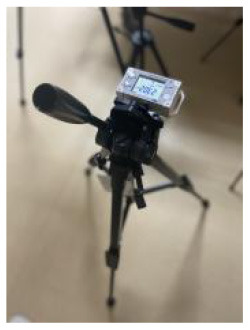
		−20 to 70°C (temperature)	±0.20°C: 0–50°C		
		0–100% (relative humidity)	±2.5% RH		
Indoor air quality	Sniffer4D mapper	0~1000μg/m^3^ (PM1.0, PM2.5, PM10)	1μg/m^3^	1 s	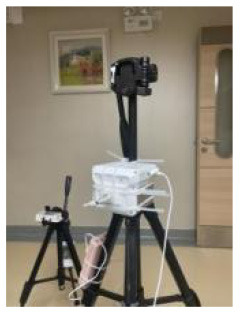
		0~11ppm (NO_2_)	<1.1ppb		
		0~11ppm (CO)	<0.6ppb		
		0~15ppm (SO_2_)	<0.8ppb		
Outdoor environment	2000 series WatchDog weather station	−40 to 125°C (air temperature)	±0.4°C at −40 to 90°C	1 min	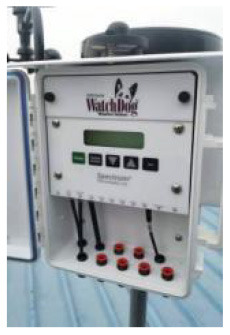
		0%-100% (Relative Humidity)	±0.2%RH at 25°C		
		0–1,500 W/m^2^	±5%		

### Measurement settings

**Figure 4:**
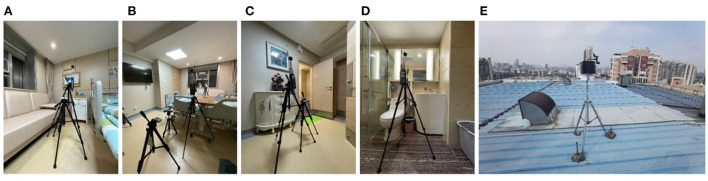
Instruments' position, real scene. **(A)** Rest area; **(B)** Reception area; **(C)** Lobby; **(D)** Toilet; **(E)** Roof.

The analysis based on ASHRAE Standard 55, Building Thermal Environment Test Method Standard JGJ/T 347, Civil Building Lighting Design Standard GBJ 133, Acoustic Environment Quality Standard GB 3096, Indoor Air Quality Standard GB/T 18883 and other approaches showed that these standards correspond to environmental measuring instruments. There are different requirements for placement position, quantity, and height. In this study, the instrument's positioning considered older adults. The geometric center point of the small space and the measurement points were arranged in six rows along the long axis as the instrument placement point. These were located in areas that do not receive direct solar radiation. The height is 0.6 and 1.2 m above the ground, depending on the position of the older adults. When the older adults are seated, the instrument is located at 0.6 m. When they stand, it is located at 1.2 m. Each instruments should be at least 1.0 m away from the walls and windows. Some instruments require flexibility based on occupant activities or comparative data. The device was stabilized for 10 min and recorded measurements for ~2 days (Figures 3, 4).

## Results

### Outdoor environment

**Figure 5:**
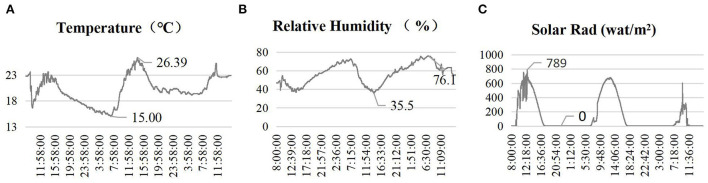
Outdoor environment. **(A)** Temperature; **(B)** Relative humidity; **(C)** Solar rad.

Based on the valuable data obtained by the 2000 Series WatchDog weather station monitoring the building roof's outdoor weather parameters for 2 days, some results were accepted after sorting and analyzing. The measured data for the outdoor physical environment are shown in Figure 5. From December 13–15, 2021, the outdoor temperature, relative humidity, and solar radiation changed periodically. The maximum outdoor temperature was 26.39°C at 1:30 p.m. and the minimum temperature was 15°C at 1:30 p.m. The temperature difference was thus more than 11°C, and it must be accounted for through adjustment measures, such as long-sleeved clothes or doors and windows. The highest relative humidity was 76.1% at 6:00 a.m. and the lowest was 35.5% at 4:00 p.m. Studies have shown that most people feel more comfortable with a relative humidity of 30–80%. Therefore, the humidity of the outdoor environment reached a comfortable standard. The solar radiation reached 789 wat/m^2^ at noon, and the lowest recording was 0 wat/m^2^. The family ward is located in southern China, which has a humid and cold climate.

### Evaluation of the thermal environment

The temperature and humidity data of the six measurement points in the family ward in this study were obtained through actual measurement (Table 3). The monitoring results showed differences in the temperature and humidity obtained by different measurement instruments, and the data from other measurement points also showed consistent variation. The testers were mainly active at measurement points 1 and a. Based on the results, the temperature and humidity data of these two measurement points were generally higher than those of the other measuring points. It is possible that some of the data were affected by the tester's mobile debugging instrument, such as measurement point 3. The temperature fluctuation range of each measuring point was about 2–3°C. Furthermore, the maximum temperature was below 25°C, and the minimum temperature was controlled above 20°C. The humidity fluctuation range of each measurement point was maintained at 12–13%. The maximum humidity was below 55% and the minimum was above 40%. From the test data, the indoor temperature and humidity of the family ward in this study were within a comfortable range. Whether it is suitable for older adults with different physical conditions requires follow-up experimental support.

**Table 3:** Measured results of thermal environment elements.

** 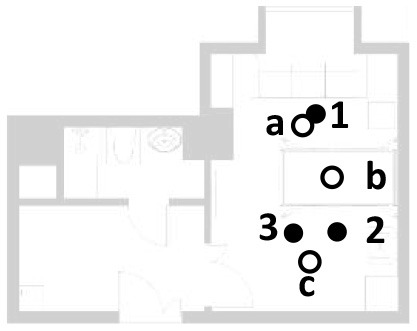 **	**Measuring instrument: HOBO-MX2301A**			**Measuring instrument: HOBO-MX1104A**		
	**Measurement** **point 1**	**Measurement** **point 2**	**Measurement** **point 3**	**Measurement** **point a**	**Measurement** **point b**	**Measurement** **point c**
Relative humidity (%), MAX	54.10	53.78	54.37	56.77	55.99	55.74
Relative humidity (%), MIN	42.68	41.87	42.49	43.37	42.27	42.31
Temperature (°C), MAX	23.49	23.66	23.49	23.57	23.36	23.41
Temperature (°C), MIN	21.28	21.47	21.50	20.92	21.48	21.63

**Figure 6:**
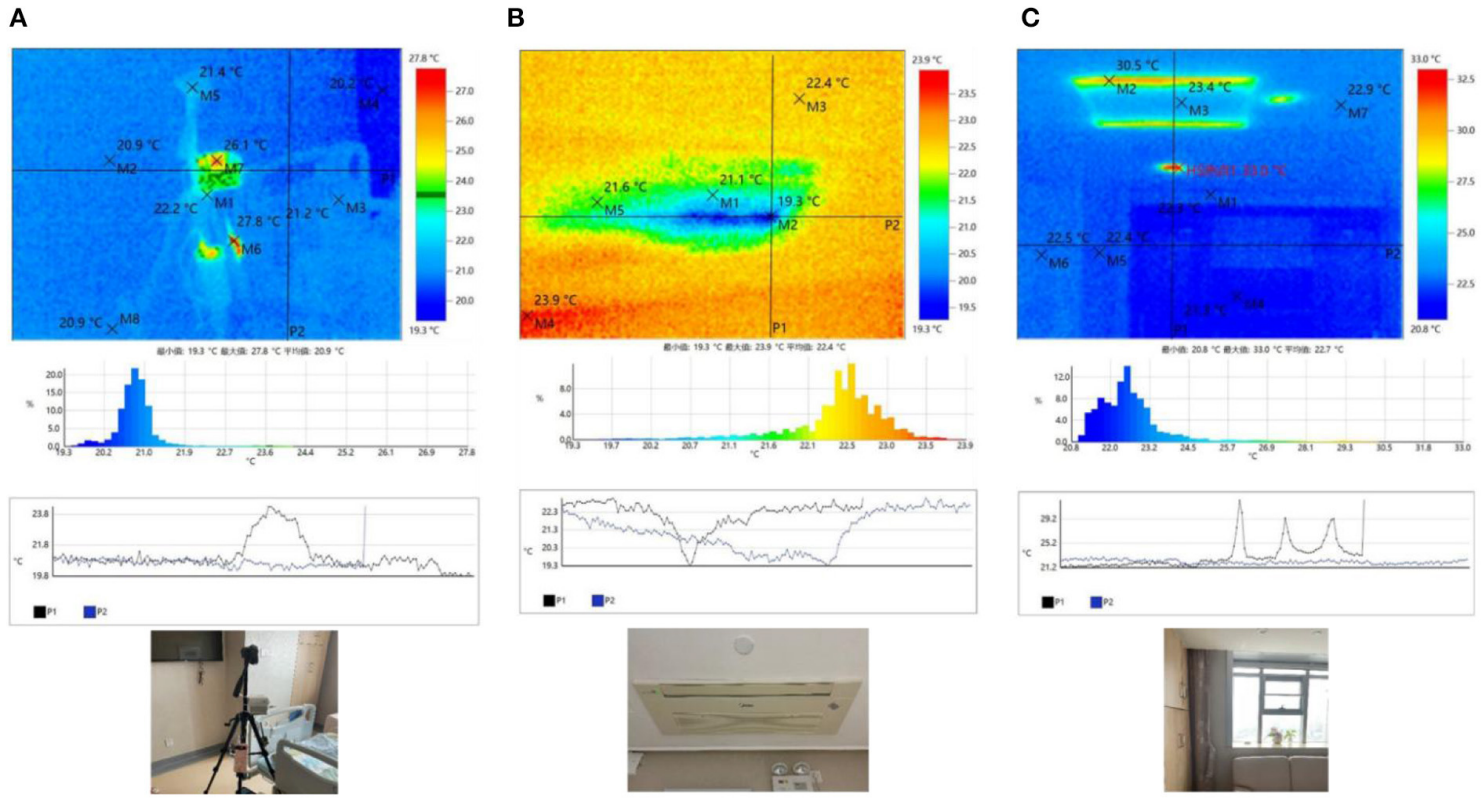
Surface temperature. **(A)** Bed area; **(B)** Air conditioning area; **(C)** Window area.

**Figure 7:**
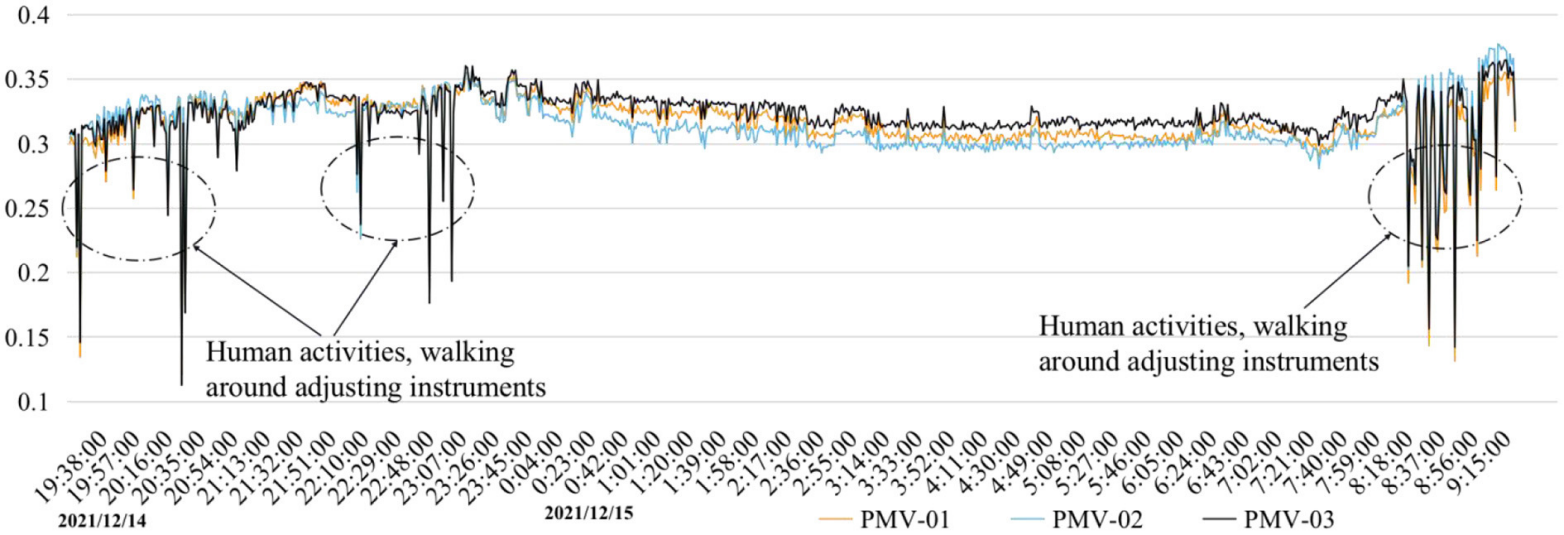
Main area of activity measurement points and calculated PMV value.

The thermal imager recorded the inner surface temperature of the internal walls, windows, and furniture of the family ward. Some of the measurement results are shown in Figure 6. The surface temperature of the air quality measuring instrument reached the commanding height of the surface temperature of the hospital bed area, locally reaching 27.8°C. For other indoor walls, it was between 20 and 22°C. In the indoor ceiling section, the air conditioning system contributed to a lower temperature, as low as below 20°C. The indoor network signal monitoring device contributed a temperature of 23.9°C. The roof temperature was 22.4°C. In the area of the room with windows and lamps, the temperature of the visible lights was as high as 33.0°C, the minimum temperature of the window glass was 20.8°C, and the other walls were all above 22°C. The above data analysis shows that the indoor temperature of the family ward in this study was within the standard range but was slightly cold. The indoor wall temperature was consistent with the average indoor air temperature measured by the measurement instrument. Therefore, the mean radiation temperature in the family ward is reflected by the temperature of each surface. The specific positions of measurement points 1, 2, and 3 are shown in Figure 6. The indoor PMV was calculated based on the actual measured environmental parameters, and the corresponding thermal environment index values at different time points were obtained (Figure 7). The PMV was between −0.5 and +0.5, indicating an overall comfortable and good thermal environment index within the family ward. The sudden change in some data was mainly caused by the experimenter's debugging of the instruments.

### Evaluation of the acoustic environment

**Figure 8:**
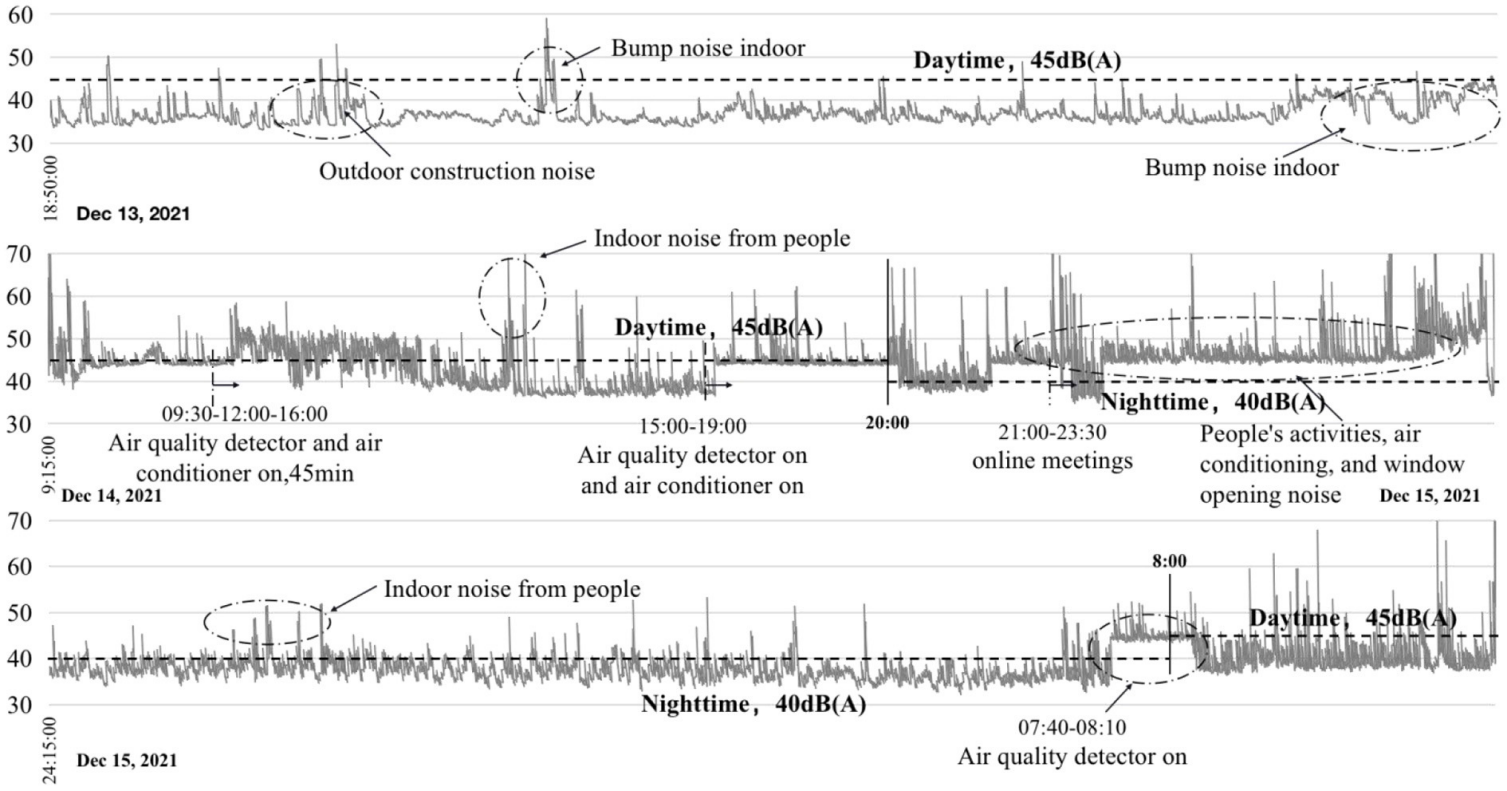
Recorded noise values from December 13 to 15.

Different interference measures in different periods affected the indoor acoustic environment of the family ward. As shown in Figure 8, the indoor acoustic environment exhibited irregular fluctuations. For example, three unfavorable noise domains appeared on the evening of December 13, when the doors and windows were closed. The first was caused by the closing of the outdoor construction site at night. Despite the doors and windows acting as barriers, the noise value still exceeded the recommended limit. The second and third were due to the routine activities of laboratory personnel. On December 14, there were two more constant noise values, both due to the activities of laboratory personnel. For example, the experimenter turned on the air quality monitoring instrument, the ventilation mode of the air-conditioning system, and the TV. The two noises that exceeded the standard on the morning of December 15 were also caused by indoor personnel activities and the adjustment of the indoor testing equipment.

Due to the activities of indoor occupants and outdoor urban dwellers, certain incidental factors can affect the results during a sound test. The noise level in this study for the entire day of December 14 was slightly higher than the specified value. Some abnormal sound levels are 55 dB (A) to 70 dB (A). From December 13 to 15, although there were still abnormally fluctuating noise values during this period, the overall sound environment was good and met the standard requirements. It is worth noting that the higher noise values that appear for a short period can be reduced by taking appropriate measures in subsequent renovations.

### Evaluation of the lighting environment

**Figure 9:**
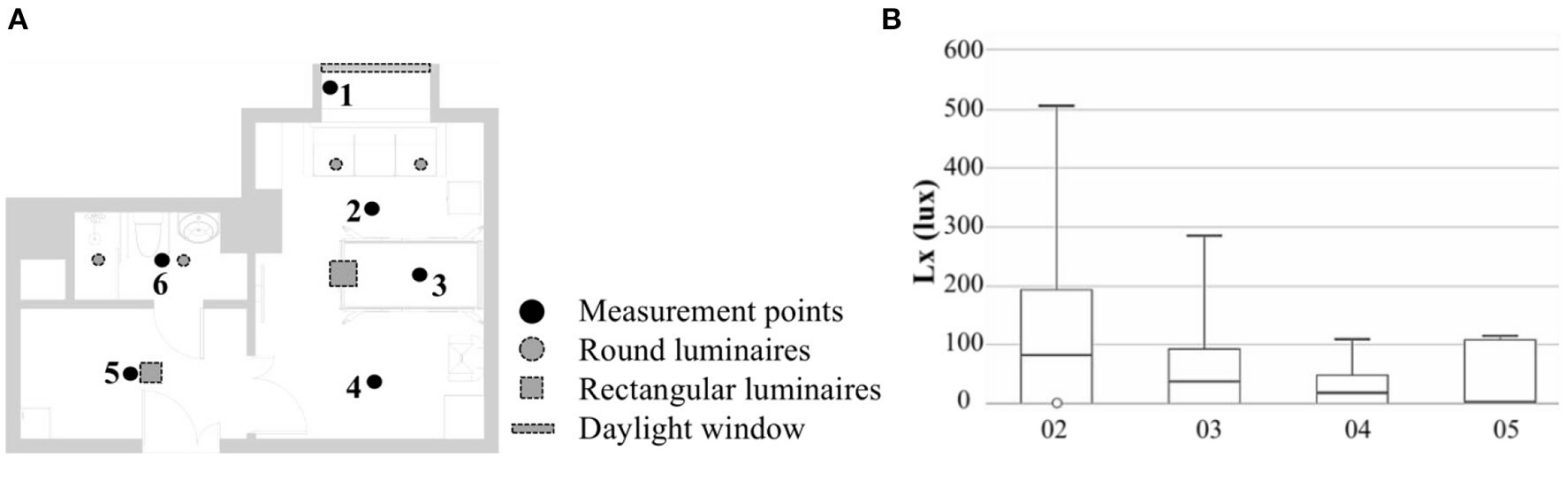
Lighting environment measurement points. **(A)** Point positions; **(B)** Illuminance intervals.

In this study, we selected six measurement points in the family ward (Figure 9). Measurement point 1 was a comparison test point on the windowsill. Measurement point 2 was located in the sofa activity area, measurement point 3 above the hospital bed, measurement point 4 in the reception area, measurement point 5 in the hall, and measurement point 6 in the bathroom. Measurement points 2 and 3 were in the bedroom area with a partial reading function; the mixed lighting thus should meet the requirements of 100–300 lx, and the natural lighting should meet the requirements of at least 300 lx. Measurement point 4 was located in the living room and needed to meet the mixed lighting standard value of 200 lx and a natural lighting illuminance value of 300 lx. The passage of measurement point 5 needed to meet the requirements of a 100 lx mixed lighting intensity. Measurement point 6 was located in the bathroom and needed to meet the illuminance requirement of 100 lx for mixed lighting.

**Figure 10:**
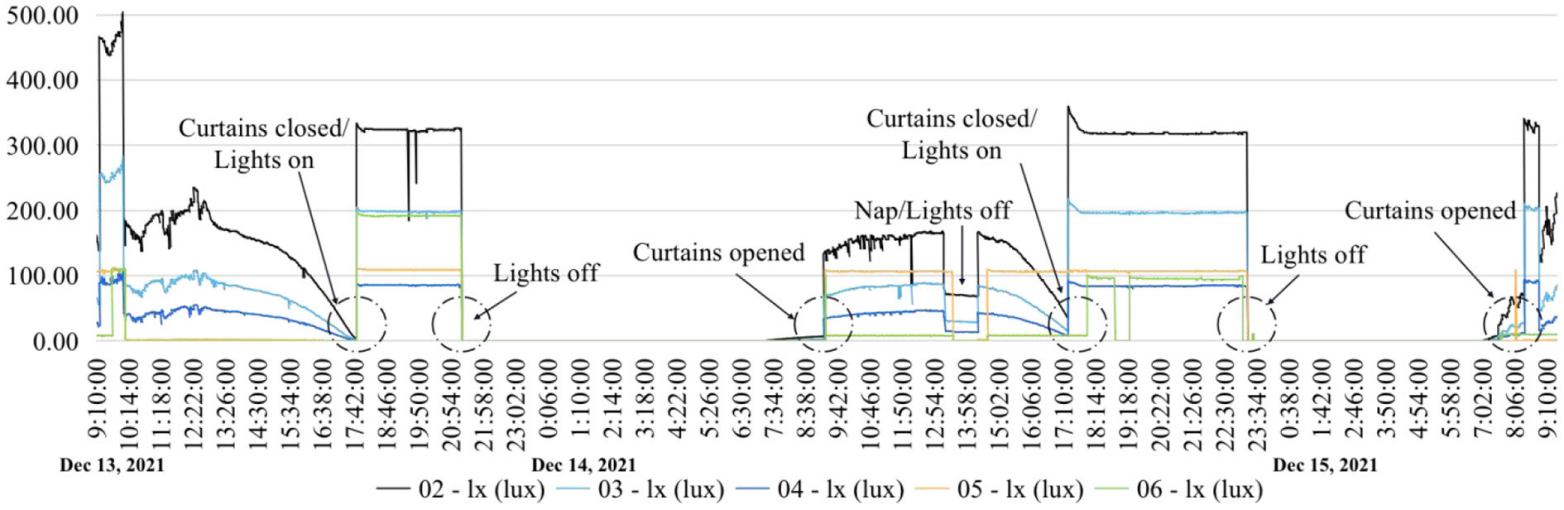
Recorded lighting values from December 13 to 15.

Figure 10 shows the three set working conditions: working condition 1 with artificial lighting turned on and the curtains open, working condition 2 with artificial lighting turned off and the curtains opened, and working condition 3 with closed curtains and artificial lighting turned on. Based on the results of the three working conditions, measurement point 2 met the standard requirements of mixed lighting, but its natural lighting illuminance still needed to be increased by 100 lx. The mixed and natural lighting at measurement points 3 and 4 did not meet the standard requirements. Measurement point 5 met the requirements with the assistance of artificial lighting, but the natural lighting in this area was poor. Measurement point 6 relied entirely on artificial lighting, which met the illumination requirements, but the area lacked natural lighting and thus needs improvement. In general, the quality of the light environment in the family ward was poor and needed to be improved.

### Evaluation of the indoor air quality

In this study, a measurement control group was established to analyze indoor environmental quality (Table 4). The measurement started at 9:00 a.m. on December 13, and there was one participant in the room. This study selected a control group for discussion. From 17:00 to 17:45 on December 13, the working conditions were to open the windows for ventilation, introduce fresh air, and turn off the air conditioner. Another set of control experiments was conducted from 17:00 to 17:45 on December 14. The working conditions were to turn on the air conditioner in the two-grid ventilation mode and close the doors and windows.

**Table 4:** Recorded values of indoor air quality elements.

** 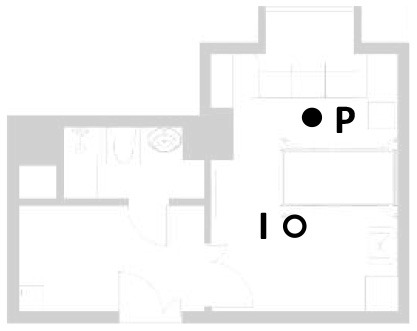 **	**Measurement instrument: Sniffer4D mapper (P: experimenter, 1 person; I: Instrument location)**					
	**SO2 μg/m^3^**	**CO mg/m^3^**	**NO2 μg/m^3^**	**PM1.0 μg/m^3^**	**PM2.5 μg/m^3^**	**PM10 μg/m^3^**
Dec. 13, 17:00–17:45 (Average value)	6.75	0.97	67.94	23.79	37.99	41.43
Dec. 14, 17:00–17:45 (Average value)	8.72	1.34	71.29	29.43	47.28	51.54
Standard limit value (Average value)	1 h ≤500 μg/m^3^	1 h ≤10 mg/m^3^	1 h ≤240 μg/m^3^	–	24 h ≤150 μg/m^3^	24 h ≤100 μg/m^3^

**Figure 11:**
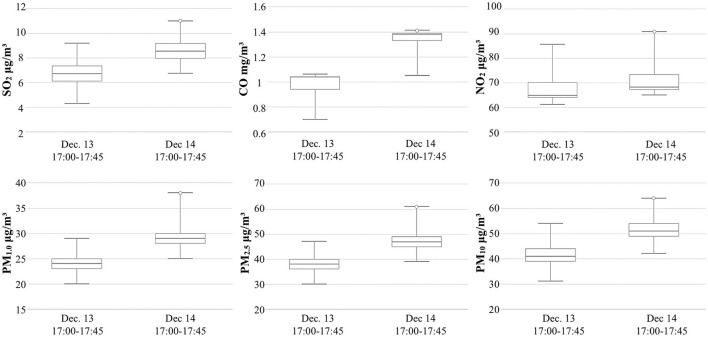
Comparison of indoor air quality elements.

Figure 11 shows the content levels of the various elements in the indoor environment of the family ward. In general, the content levels of each element on December 14 were significantly higher than those on December 13. The SO_2_ content level was 2 μg/m^3^ higher, the CO content level was 0.35 mg/m^3^ higher, the NO_2_ content level was 4 μg/m^3^ higher, the PM_1.0_ content level was 5 μg/m^3^ higher, the PM_2.5_ content level was 4.5 μg/m^3^ higher, and the PM_10_ content level was higher than 10 μg/m^3^. Thus, the CO content increased most dramatically.

## Discussion

### Summary

Through experimental analysis, it was concluded that the occupants' behavior affects the quality of the indoor physical environment and directly affects the comfort and health of the occupants. Therefore, allowing occupants to control the indoor physical environment during the supercooling period, such as by heating, cooling, and introducing fresh air, should be considered in the designing of a family ward. It is important to note that in residential environments, occupants' environmental preferences and regulatory behaviors vary widely, especially among older adults, and buildings must be designed to accommodate these changes while simultaneously increasing building energy use and reducing emissions. These issues are significant in the context of climate change and rapid aging.

This study found that family wards performed better in terms of thermal environment and indoor air quality. However, some improvements to the light and acoustic environments are needed. In addition, the living habits of the occupants, especially older adults, directly affect the quality of the indoor environment. To improve the livability of the indoor environment of family wards, this study puts forth several optimization suggestions.

- There are abundant international standards and documents across societies, and the purpose of optimizing the physical environment is achieved by strictly limiting the standard values of various physical environment parameters. However, considering the living environment in China, resident characteristics, and the habits of adjusting to the environment, regional features should be taken into account in the design of a family ward. We compared the test results of this study with Chinese and international standards. This study aims to achieve a more scientific evaluation of the quality of the physical environment in family wards. For example, according to ASHRAE research on acoustic environments, the maximum appropriate noise level in a family ward ranges from 39 dB (A) to 44 dB (A). If the room is small and private, the maximum noise level should be 39 dB (A) to 48 dB (A). Moreover, the maximum appropriate noise level is 35 dB (A) to 39 dB (A) for bedrooms and 39 dB (A) to 48 dB (A) for living rooms. The ASHRAE research results are somewhat different from the Chinese standards. The Chinese standards state that bedrooms should be ≤45 dB (A) during the day and ≤37 dB (A) at night, and the living room should be ≤45 dB (A) throughout the day. Therefore, based on Chinese standards, in this study, the acoustic environment quality of wards was judged according to the provisions. The same applies to other physical environmental factors, as explained above.
- The family ward met the general comfort standard and performed well in terms of the thermal environment. To optimize the thermal environment of the family ward examined in this study, we first considered preventing the room from overheating or overcooling. The cooling and heating functions of the air-conditioning system were not used in this experiment; therefore, the results obtained showed a slightly colder state at night. In optimizing the ward's physical environment in the future, the heat source and cooling system can be effectively controlled so that the indoor physical environment can be adjusted to a temperature suitable for older adults' thermoregulation. In addition, fresh air can be introduced by adjusting the temperature and moisture content of each surface in the room, while enhancing the natural ventilation rate. We deal with thermal comfort under different control modes, and alternative methods can be selected, such as correcting the clothing value by changing the occupants' clothing to from a level at which they feel uncomfortable (such as 1.0 clo) to a relatively comfortable level (such as 0.5 clo).
- Considering the existing standard values for the acoustic environment of residential buildings in China, limits of 45 dB (A) during the day and 40 dB (A) at night were suggested. From this study, it can be observed that the indoor acoustic environment is influenced by occupant behavior. It is also significantly influenced by outdoor-specific sound sources (e.g., traffic noise, outdoor construction noise, adjacent dwellings), home technical installations, and occupants' ability to adjust these factors. Considering the family ward's particular function room and considering the physical characteristics of older adults, from this perspective, the investigated family ward needs to strengthen its noise control. In particular, at night, the outdoor ambient noise needs to be eliminated. In indoor acoustic environments, there are many optimization strategies, such as, applying sound insulation materials to reduce the noise in the living environment as much as possible, using doors, windows, and other enclosures with good sound insulation effects, and the use of soft indoor decoration that adjusts indoor reverberation time and weakens noise intensity. In addition, noise sources should be avoided, such as reducing the noise generated by indoor equipment.
- Optimizing the indoor light environment, on the premise of meeting the standard requirements should focus on the weakening physical functions of occupants with age as well as the occupants' need for high-quality light environments. In this study, the quality of the light environment in the family ward was poor, and the requirements of relevant standards could only be met when natural and artificial lighting were used in conjunction. The color, temperature, and warmth of the light environment of the family ward in this study need to be further investigated. Based on the current experimental results, we first need to strengthen the illuminance standards of natural and artificial lighting, achieve a better lighting state, and improve the overall light environment quality of the ward with different light color effects. Many specific approaches can be employed to optimize the physical environment of this family ward. Examples include using illuminance that matches the visual function of older adults, adjusting the intensity of sunlight during different seasons, quantifying the measurement of visible light passage, and using movable visors and window glass to control color rendering. In addition, artificial lighting can be used to supplement natural lighting when necessary. An indoor mixed-light environment can satisfy the standard requirements with the inclusion of high-performance lamps. In winter, or when natural lighting is insufficient, consideration should be given to incorporating artificial lighting environments.
- After 2 days of actual measurement, the indoor environment of the family ward was of good quality and fully met the limits specified by the standards. Regarding air quality, the environment can be optimized for elements that have detrimental effects on human health and comfort. Specific measures included filtering the air coming in from the outdoors, increasing the frequency of natural ventilation and the ventilation rate of mechanical ventilation, increasing the purification effect of green plants, and choosing low-polluting home improvement materials. When occupants living alone, opening windows for ventilation can bring better indoor environmental quality than turning on an air conditioner. This study involved the observation of the changes in indoor air quality brought about by occupants' adjustment to the indoor environment. When designing a healthy family ward environment, designers must also consider the environmental impact of building materials and functional layouts. Simultaneously, the focus is on the possibility of occupants adapting to these conditions.

### Limitations

In the future, people, especially older adults, will have higher requirements for the indoor physical environments in their daily lives. This study lacks systematic, scientific, and reasonable guidelines for optimizing the interior environment of existing residential buildings and appropriate design guidance for interior space design. The four physical environment elements—the acoustic environment, light environment, thermal environment, and indoor air quality—involve considerable content. This study made some attempts in terms of research, but more scholars need to discuss these issues as key research objects. Based on the current research data, the home-based care environment in South China needs to be optimized. The environmental needs of the different types of rooms for older adults are poorly understood. For example, in the bedroom, the acoustic, thermal, and light environments interact more closely with older adults' health. Indoor air quality may be a more important factor for older adults in living rooms. Therefore, studying the interaction between the indoor physical environment and the behavioral habits and health status of older adults is of great importance for improving their quality of life.

The limitations of this study are that the sample size of the family ward was insufficient, and the total time of the physical environment test was inadequate. The research still requires the participation of older adults as an experimental subject to better support the research results. It is important to note that this study did not measure these factors during a time outside the supercooling period; therefore, the measurements were not well validated. Although the supercooling period was selected for measurement, it did not represent the environmental conditions of the family ward during winter and summer. Fortunately, the family ward has been renovated for older adults, and it plans to admit older adults as the next step, laying a good foundation for follow-up research. In addition, this study has certain limitations in terms of benchmarking. Owing to the lack of international discussions on the research object of family wards, there are no building norms and standards directly aimed at the physical environment of this research object. This study is based on the consideration of older adults and individuals with disabilities, combined with the relevant content of the norms and standards of hospital buildings, residential buildings, green buildings, and healthcare buildings. In the future, as scholars focus more on family wards and advance research on its physical environment, it is expected that this research and subsequent discussions will contribute to the specifications and standards for the physical environment of these family wards.

## Conclusions

This study presents the results of a short-term physical environment monitoring project in a Guangzhou family ward. Our research provides a comprehensive understanding of the current state of indoor and outdoor environments during the supercooling period using measured data and software to determine the indoor and outdoor physical environments of family wards. Our findings suggest that occupant activity directly affects the indoor physical environment. This is primarily reflected in the control activities of the occupants. During the supercooling period, by adjusting the opening and closing curtains and turning lamps on and off, adjusting windows and air conditioning systems, and adjusting the activity types of indoor functional areas, methods to improve the quality of the indoor physical environment can be discovered and harnessed to relieve discomfort.

The study also found that traditional indoor environmental quality judgments are mostly subjective judgments based on vision, hearing, and smell, which are usually inaccurate. This study combines subjective and objective measurements and software analysis with personal judgment to achieve a scientific study of the indoor physical environment and propose a reasonable optimization path. Taking internationally recognized standards as the research background, this subjective and objective analysis of the environment will help the occupants, especially older adults, better understand their environment and inform future indoor environment optimization measures.

Although this study has certain limitations, the analytical methods used here have general implications in terms of artificially setting different indoor environment control modes, comprehensive interpretation, and the analysis of the changing characteristics of the four elements of the indoor physical environment. This study focuses only on family wards during the supercooling period. A similar method will be used at other times of the year to conduct control experiments. By observing the characteristics of the interactions between people and the environment, we can summarize the paths that optimize the indoor physical environment. This study provides guidance for environmental modification and for the living habits of future occupants' during the supercooling period.

This study found that there is currently a lack of research on the interaction mechanism between occupants' living patterns and elements of their living environment, and the proportion of older adults among the subjects of sustainable environmental research remains to be assessed. Our study is the first step toward bridging this gap. Future research will address the issue of environmental health by exploring the impact mechanism of older adults' living behaviors in different seasons throughout the year on elements of their physical environment. This research has implications for the health of older adults, sustainable environments, healthy cities, and policy and suggests introducing better indoor physical environment requirements for family wards to optimize their indoor environmental parameters, prevent health disorders, and improve the quality of life of older adults.

## Data availability statement

The raw data supporting the conclusions of this article will be made available by the authors, without undue reservation.

## Author contributions

YZ and XL: conceptualization. XL: methodology, formal analysis, and funding acquisition. YZ: software, resources, and data curation. XL, QM, and LC: validation. YZ and BL: investigation and visualization. YZ, XL, and BL: writing—original draft preparation and writing—review and editing. QM and LC: supervision. All authors have read and agreed to the published version of the manuscript.

## Funding

This research is supported by the National Key R&D Program of China (Grant No. 2021YFC2009400); the National Natural Science Foundation of China (Grant No. 52108011); Department of Education of Guangdong Province (Grant No. 2021KTSCX004); Department of Housing and Urban–Rural Development of Guangdong Province (Grant No. 2021-K2-305243); Science and Technology Program of Guangzhou, China (Grant No. 202102020302); Guangzhou Philosophy and Social Science Planning 2022 Annual Project (Grant No. 2022GZQN14); the Fundamental Research Funds for the Central Universities (Grant No. QNMS202211); State Key Laboratory of Subtropical Building Science, South China University of Technology (Grant No. 2021ZB16); China Postdoctoral Science Foundation (Grant No. 2021M701249). This work was also supported in part by the scholarship from the China Scholarship Council (CSC) under the CSC Grant Nos. 202106150080 and 202006150053.

## Conflict of interest

Author XL was employed by Architectural Design and Research Institute Co., Ltd. The remaining authors declare that the research was conducted in the absence of any commercial or financial relationships that could be construed as a potential conflict of interest.

## Publisher's note

All claims expressed in this article are solely those of the authors and do not necessarily represent those of their affiliated organizations, or those of the publisher, the editors and the reviewers. Any product that may be evaluated in this article, or claim that may be made by its manufacturer, is not guaranteed or endorsed by the publisher.
